# Salvationism and the Care of the Sick

**Published:** 1892-02-20

**Authors:** 


### The Hospital

A Weekly Journal of

Science, Medicine, Nursing, and Philanthropy.

*For Hospitals, Asylums, Infirmaries, Dispensaries, Medical Practitioners, Students, Nurses, and the Charitable Public.*

Vol. XI.—No. 282.] [Feb. 20, 1892.

### SALVATIONISM AND THE CARE OF THE SICK.

General Booth is the man of the hour. He and his officers know how to fill the world with their presence. They have carried the art of advertising to a high pitch of success. When a man has attained to such a position as that of the General in public estimation, criticism often hesitates to touch him. Some critics, awed by the magnitude of the phenomenon presented to them, and impressed by the evident effect produced upon the multitude, will not speak, lest they should bring a hornet's nest about their ears. Others, thinking that the organisation known as the Salvation Army has got quite out of hand like a runaway horse, give up the attempt at criticism in a kind of despair. Neither of these courses is manly, English, or right. The greater, the more startling, and the more effective is a given organisation, the more necessary is it, in the interests of the human beings whom it concerns in so many ways, to look at it fairly and squarely, to look at it often, and to speak of it without fear or favour.

Salvationism is a form of religion. The care of the sick poor is also a form of religion. Perhaps it would be more critically accurate to say that Salvationism is a department of religion rather than a form of it. Nobody, except perhaps the very crudest of Salvationists, would say that General Booth and his preaching disciples deal in any sense with religion as a whole. They are associated with a large organisation, and their principal function is to perform the part of recruiting sergeants for that organisation. When a man has once been persuaded to become a member of the Salvation Army he has been placed in a certain position of religious instruction and religious possibility. He has, in fact, been introduced into a religious elementary school, and is a more or less apt and docile pupil. But it cannot be said that he is any more than that. He may, from natural incapacity or indolence, never get beyond the pupillary stage; or he may, and in many cases he actually does, first form a habit of playing truant, and finally abandons the elementary school of Salvationism altogether.

Now, the Salvation Army, as an elementary school of religion, has undoubtedly a definite value—the value of an elementary school. That value will be considered great or small according to the standpoint of the person who makes the valuation. A man who looks upon elementary religious schools as the most valuable of all institutions will naturally set a high worth upon the Salvation Army. But the man of more comprehensive mind perceives that there are many other institutions in the world which are as essential to its physical and mental, and even to its spiritual well-being as elementary religious schools. There is, for example, the study of justice between man and man, and of the law that is based thereupon. There is the question of the health of men's bodies in unfavourable climates, and in injurious surroundings and circumstances. There is the science of religion in its less elementary forms, and in its wider outlook—religion which must reconcile itself to the discoveries of science, the wide-reaching enquiries and speculations of keen and philosophic minds, and to the moral necessities and difficulties of those who have to play their daily part among the most strenuous and the most versatile of the intellects of the time.

It is plain, then, that the world does not consist entirely of the Salvation Army, and those who are food for its powder. There are other things to think of than it; and there are other necessities to provide for than those which it may legitimately plead. Religious men, particularly those of the more impressionable sort, often have generous hearts that yield to the loudest importunity. But in this country most religious men are Christians; and Christianity is not, and was never intended to be, a religion of mere impulse and fanaticism. It was intended, first and above all things, to be a practical divine life. The Founder of Christianity did not, on any recorded occasion, appeal to the cruder and coarser instincts of those who listened to Him. His appeal constantly was to the higher reason and nobler manhood of the people.

The *Daily Graphic* and other illustrated papers have presented us with pictures of General Booth triumphantly moving up the Solent on board his sea-going vessel, and reviewing thousands of his admirers as they circled round him on board other vessels chartered for the purpose. Well, there is not, perhaps, any very great harm in this; but neither, on the other hand, is there any very great good. The mere scientific study of the founder of Christianity and his work, impels one to ask who it was that said, "the kingdom of heaven cometh not with observation; neither shall ye say, lo here, or lo there, for the kingdom of God is within you." One cannot but feel that a man who had drunk deeply into the spirit of the incomparable Christ would not have wished for all this parade of home coming. Even Paul when he separated from his weeping children, as the ship hove in sight which was to carry him away for ever, only "kneeled down on the shore and prayed." The scientific student of Christianity, much as he may esteem General Booth for certain important services he has rendered to public morals, cannot but feel that if Salvationism commends a small portion of the religion of Christ to one class of people it tends to make that religion look much less beautiful and noble in the eyes of another and equally important class. One could wish, indeed, on many grounds that the "Booth" personality might become less and less, and that intelligent religious principles and practices might become more and more.

The needy and the helpless appealed to the founder of Christianity as no other class did. We have not space to cite many instances. But the woman suffering from hæmorrhage who touched the hem of His mantle, and the cry of blind Bartimæus, which was responded to with so much simple dignity, may serve to show the temper of the mind of Jesus towards physical suffering and need. He attended to these people without delay, and He gave them what cost Him much. For even when His garment was but touched He "perceived that virtue had gone out of Him." We do not say that it is not of primary importance to teach religion, even in a crude and elementary form. But we do say that whilst religion is taught it should also be intelligently practised. Nay, the Founder of Christianity Himself has plainly showed that if a choice is to be made between the preaching and the practice of Christianity, the practice is emphatically to be preferred. In that simple sketch of the summing up of men's lives at a final day of account this wonderful teacher presents us with a picture of certain religious persons appearing before the Judge, and saying, in tones of remonstrance, "Lord, Lord, have we not prophesied in Thy name, and in Thy name have cast out devils, and in Thy name have done many wonderful works?" But the Judge replies, "Depart from me, I never knew you." On the other hand, a peculiar benediction is pronounced upon certain persons who had manifestly done nothing in the way of teaching and preaching religion. "Come ye blesssd, inherit the kingdom; for I was hungry and ye fed me, sick, and ye ministered unto me." "When?" ask these simple, practical folk. "Inasmuch as ye have done it to one of the least of these ye have done it unto Me."

It has seemed quite necessary to address a special article to persons who profess Christianity at this particular moment. We do not say that General Booth and his schemes should not be supported; but we do say that the sick poor should be succoured and cared for, and we express the emphatic conviction that a Christian person who cannot both succour the sick poor and support General Booth should, at least, provide for the sick poor whether he provides for General Booth or not. The men, the women, and the hapless children who crowd our hospital wards can organise no demonstrations. No trumpets ever sound for them, as they lie with bandaged limbs or in tossing fever on the beds which charity supplies. Where is Christianity seen at its best? Amid the roll of drums on the Solent, or ministering refreshment and consolation to the sick, the helpless, and the dying? Let us support the public ministrations of religion by all means. A State without religion is like a man with a brain, but without a heart. But if religion be known only in its public ministrations, and not daily carried out in every department of life, it becomes, instead of a pulsating heart vitalising the body, a mere coffin that shuts out the air of heaven, and suffocates the living as well as encloses the dead.

